# Overexpression of claspin promotes docetaxel resistance and is associated with prostate‐specific antigen recurrence in prostate cancer

**DOI:** 10.1002/cam4.4113

**Published:** 2021-07-09

**Authors:** Takashi Babasaki, Kazuhiro Sentani, Yohei Sekino, Go Kobayashi, Quoc Thang Pham, Narutaka Katsuya, Shintaro Akabane, Daiki Taniyama, Tetsutaro Hayashi, Masaki Shiota, Naohide Oue, Jun Teishima, Akio Matsubara, Wataru Yasui

**Affiliations:** ^1^ Department of Molecular Pathology Graduate School of Biomedical and Health Sciences Hiroshima University Hiroshima Japan; ^2^ Department of Urology Graduate School of Biomedical and Health Sciences Hiroshima University Hiroshima Japan; ^3^ Department of Pathology Kure Kyosai Hospital Federation of National Public Service Personnel Mutual Aid Associations Hiroshima Japan; ^4^ Department of Urology Graduate School of Medical Sciences Kyushu University Fukuoka Japan

**Keywords:** cell cycle checkpoint, claspin, DNA damage repair, docetaxel resistance, prostate cancer

## Abstract

Although docetaxel (DTX) confers significant survival benefits in patients with castration‐resistant prostate cancer (CRPC), resistance to DTX inevitably occurs. Therefore, clarifying the mechanisms of DTX resistance may improve survival in patients with CRPC. Claspin plays a pivotal role in DNA replication stress and damage responses and is an essential regulator for the S‐phase checkpoint. *CLSPN* is an oncogenic gene that contributes to tumor proliferation in several human solid tumors. However, the clinical significance of claspin in prostate cancer (PCa) has not been examined. The present study aimed to elucidate the role of claspin and its relationship with DTX resistance in PCa. We immunohistochemically analyzed the expression of claspin in 89 PCa cases, of which 31 (35%) were positive for claspin. Claspin‐positive cases were associated with higher Gleason score, venous invasion, and perineural invasion. Kaplan–Meier analysis showed that high claspin expression was related to poor prostate‐specific antigen (PSA) relapse‐free prognosis. In a public database, high CLSPN expression was associated with poor PSA relapse‐free prognosis, Gleason score, T stage, lymph node metastasis, CRPC, and metastatic PCa. Claspin knockdown by siRNA decreased cell proliferation, upregulated DTX sensitivity, and suppressed the expression of Akt, Erk1/2, and CHK1 phosphorylation in DU145 and PC3 cell lines. Furthermore, claspin expression was much more upregulated in DTX‐resistant DU145 (DU145‐DR) than in parental DU145 cells. Claspin knockdown significantly upregulated the sensitivity to DTX in DU145‐DR cells. These results suggest that claspin plays an important role in PCa tumor progression and DTX resistance.

## Graphical abstract

Claspin overexpression was related to poor PSA relapse‐free prognosis. Claspin knockdown significantly upregulated the sensitivity to docetaxel in DU145 docetaxel‐resistant cells.

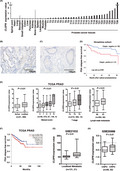

## 1 INTRODUCTION

Prostate cancer (PCa) is the most common cancer in males in many countries of the world. Additionally, it was the fifth leading cause of cancer‐related mortality in 2015.^1^ Androgen deprivation therapy (ADT) is effective in treating patients with PCa. However, once castration resistance is acquired, it will not respond to ADT, and recurrence and metastasis will occur as castration‐resistant prostate cancer (CRPC). Recently, various novel hormone treatment drugs and their combinations have appeared,^2^, ^3^, ^4^, ^5^ but docetaxel (DTX) remains one of the important taxane anticancer agents for patients with metastatic CRPC.^6^ DTX binds to polymerized microtubules and inhibits cell mitosis,^7^ and although initially effective, almost all patients with CRPC eventually become refractory. Therefore, there is a need to clarify the mechanisms related to DTX resistance in PCa.

Claspin is a nuclear protein related to DNA replication stress and damage responses and is an essential regulator for the S‐phase checkpoint.^8^, ^9^ Claspin has been found to be a factor necessary for checkpoint kinase 1 (CHK1) phosphorylation and activation by the upstream kinase, ataxia‐telangiectasia‐mutated‐and‐Rad3‐related kinase (ATR), in *Xenopus* oocyte extracts.^10^ With the ATR‐CHK1 pathway, claspin plays an essential part in maintaining replication fork stability during the S phase of the cell cycle. In the presence of DNA damage, claspin promotes ATR‐CHK1 activation, resulting in cell cycle arrest and DNA damage repair (DDR). Successful DDR promotes checkpoint recovery, but failure to repair DNA damage leads to cell death.^11^, ^12^, ^13^, ^14^, ^15^ Several genes are reported to be associated with the prognosis of PCa, such as DDR‐related molecules such as poly (ADP‐ribose) polymerase (PARP), breast cancer susceptibility gene 1/2 (BRCA1/2), and the ataxia telangiectasia mutated (ATM) gene.^16^, ^17^, ^18^, ^19^ Claspin overexpression is reported to contribute to tumor proliferation in several human solid tumors such as carcinomas of the lung, ovary, uterine cervix, stomach, and kidney.^20^, ^21^, ^22^, ^23^, ^24^, ^25^, ^26^ However, to our best knowledge, the relationship of claspin expression and its detailed function in PCa has not been analyzed previously.

Thus, the present study is the first detailed analysis of claspin in PCa, including its clinicopathological significances, biological functions, and DTX sensitivity. We performed immunohistochemical (IHC) analysis of surgically resected PCa samples and studied the association between claspin expression and various clinicopathological characteristics. We also analyzed the effect of claspin knockdown by siRNA on proliferation, DDR, and DTX sensitivity in PCa cell lines.

## 2 MATERIALS AND METHODS

### 2.1 Tissue samples

We used a retrospective study design and collected 89 primary tissue samples from patients diagnosed as having PCa who underwent prostatectomy surgery in 2012–2013 at Hiroshima University Hospital (Hiroshima, Japan). All samples were obtained with patient consent and the Ethical Committee for Human Genome Research of Hiroshima University approved the present study (approval no.: IRINHI66). For IHC analysis, only patients without preoperative ADT, radiation, and chemotherapy, and clinical evidence of distant metastasis were enrolled. Tumor staging was performed according to the American Joint Committee on Cancer classification system. Postoperative follow‐up was scheduled monthly during the first 6 months and then every 3 months after that. Serum chemistry analysis including prostate‐specific antigen (PSA) was performed at every follow‐up visit. Biochemical recurrence was defined as a PSA level of ≥0.2 ng/ml.

For quantitative RT‐PCR (qRT‐PCR) analysis, we used 28 PCa samples. All patients with the same conditions as described above were enrolled. PCa tissues were removed surgically, frozen immediately in liquid nitrogen, and stored at −80°C until use. Non‐neoplastic tissue samples (spinal cord, heart, lung, stomach, small intestine, colon, liver, pancreas, kidney, bone marrow, spleen, leukocyte, skeletal muscle, and prostate) were purchased from Clontech Laboratories, Inc.

### 2.2 qRT‐PCR analysis

Total RNA was isolated from frozen cancer tissues and cell lines using Isogen (Nippon Gene). One microgram of total RNA was converted to cDNA with a first‐strand cDNA synthesis kit (Amersham Biosciences Corp.). The qPCR was performed as described previously.^27^ ACTB‐specific PCR products, which were amplified from the same RNA samples, served as internal controls. The claspin primer sequence was forward primer AGAGCAGCCACAATAGCAGC; reverse primer ACGGCCTGTTTGTCTGTTGC.

### 2.3 Immunohistochemistry

IHC analysis was performed with a Dako Envision + Mouse Peroxidase Detection System (DakoCytomation). Antigen retrieval was performed by microwave heating in citrate buffer (pH 8.0) for 1 h. Peroxidase activity was blocked with 3% H_2_O_2_–methanol for 5 min and the sections were incubated with normal goat serum (Dako Cytomation) for 10 min to block nonspecific antibody binding sites. Sections were incubated with a rabbit polyclonal anti‐claspin antibody (ab3720, Abcam, Plc., Cambridge, UK, 1:20000) or a mouse monoclonal anti‐androgen receptor (AR) antibody (sc‐7305, Santa Cruz Biotechnology, CA, USA, 1:400) for 1 h at room temperature, followed by incubation with Envision + anti‐rabbit or ‐mouse peroxidase for 1 h. The sections were incubated with DAB Substrate‐Chromogen Solution (Dako Cytomation) for 5 min for color reaction and then were counterstained with 0.1% hematoxylin. Negative controls were created by the omission of the primary antibody.

The expression of claspin in PCa was scored in all tumors as positive or negative. When more than 5% of tumor cells were stained, immunostaining was considered positive for claspin (we set a cut‐off value of 5% because no expression above 5% was observed in non‐neoplastic areas). Two observers (T. B. and K. S.), without the knowledge of the patient's parameters, independently reviewed immunoreactions in each specimen using these definitions. A consensus review resolved any slight discrepancies or differences between the observers under a double‐headed microscope after independent consideration.

### 2.4 In silico analysis

The GEPIA web tool was used to determine *CLSPN* expression in The Cancer Genome Atlas (TCGA) prostate adenocarcinoma (PRAD) dataset.^28^ Prostate tumors were separated into high‐ and low‐*CLSPN* expression groups such that the high‐*CLSPN* expression group accounted for nearly a 50% level of the total. The expression array data were downloaded from GEO under accession numbers GSE21032,^29^ GSE35988,^30^ GSE77930,^31^ GSE104786,^32^ GSE126078,^33^ and the dataset of Abida et al.^34^ Statistical analysis using the paired *t*‐test or one‐way ANOVA was conducted with GraphPad Prism 6 (GraphPad Software, Inc.). *p* < 0.05 was considered statistically significant.

### 2.5 Cell lines

We used four cell lines derived from human PCa. DU145, PC3, and LNCaP were purchased from the Japanese Collection of Research Bioresources Cell Bank. 22Rv‐1, C4‐2, and DTX‐resistant DU145 cells (DU145‐DR) were kindly provided by Dr. Masaki Shiota (Kyushu University, Fukuoka, Japan). All cell lines were maintained in RPMI 1640 (Nissui Pharmaceutical) containing 10% FBS (Whittaker) in a humidified atmosphere of 5% CO_2_ at 37°C.

### 2.6 Western blotting

Tumor cells were lysed for Western blotting as described previously.^35^ Primary antibody, claspin (ab3720, Abcam, Plc.), Akt, phospho‐Akt (p‐Akt), p44/42 MAPK (Erk1/2), phospho‐p44/42 MAPK (p‐Erk1/2), CHK1, phospho‐CHK1 (Ser317), phospho‐CHK1 (Ser345), phospho‐Histone H2A.X (Ser139), and cleaved PARP (#4691, #4060, #4695, #9101, #2360, #2344, #2348, #9718, and #5625, respectively, Cell Signaling Technology, Inc.) were used. β‐Actin antibody (Sigma Chemical) was used as a loading control.

### 2.7 RNA interference

RNA interference was carried out to knock down endogenous claspin as described previously.^36^ siRNA oligonucleotides for claspin and negative control were purchased from Invitrogen. Transfection was performed using Lipofectamine RNAiMAX (Invitrogen) according to the manufacturer's protocol. Briefly, 60 pmol of siRNA and 10 μl of Lipofectamine RNAiMAX were mixed in 1 ml of RPMI medium (10 nmol/L of final siRNA concentration). The mixture was added to the cells after 20 min of incubation. The cells were analyzed at 48 h after transfection in all experiments.

### 2.8 Cell proliferation assays

Cell proliferation was assessed with a standard MTT assay, which detects the dehydrogenase activity in viable cells. In total, 5 × 10^3^ cells were seeded in each well of 96‐well culture plates. Then, cell proliferation was monitored after 1, 2, and 4 days. We performed five different experiments and calculated the mean and standard deviation (S.D.) in each of the MTT assays.

### 2.9 Drug treatment

DTX was obtained from Sanofi‐Aventis and handled according to the manufacturer's recommendations. The cells were treated with various concentrations of DTX for 24 h. After DTX treatment, MTT assay was performed. Drug sensitivity curves and IC50 values were calculated using Microsoft Excel.

### 2.10 Statistical analysis

Correlations between the clinicopathological parameters and claspin expression were analyzed using Fisher's exact test. Kaplan–Meier survival curves were constructed for claspin‐positive and claspin‐negative patients, and PSA relapse‐free survival rates of the two groups were compared. Differences between the PSA relapse‐free survival curves were tested for statistical significance by log‐rank test. Univariate and multivariate Cox regression analyses were used to evaluate the associations between clinical covariates and PSA relapse‐free survival as described previously.^37^ The JMP Pro 15 software program (SAS Institute Inc.) was used for statistical analysis.

## 3 RESULTS

### 3.1 Clinicopathological significance of claspin expression and prognosis for biochemical recurrence in PCa

**FIGURE 1:**
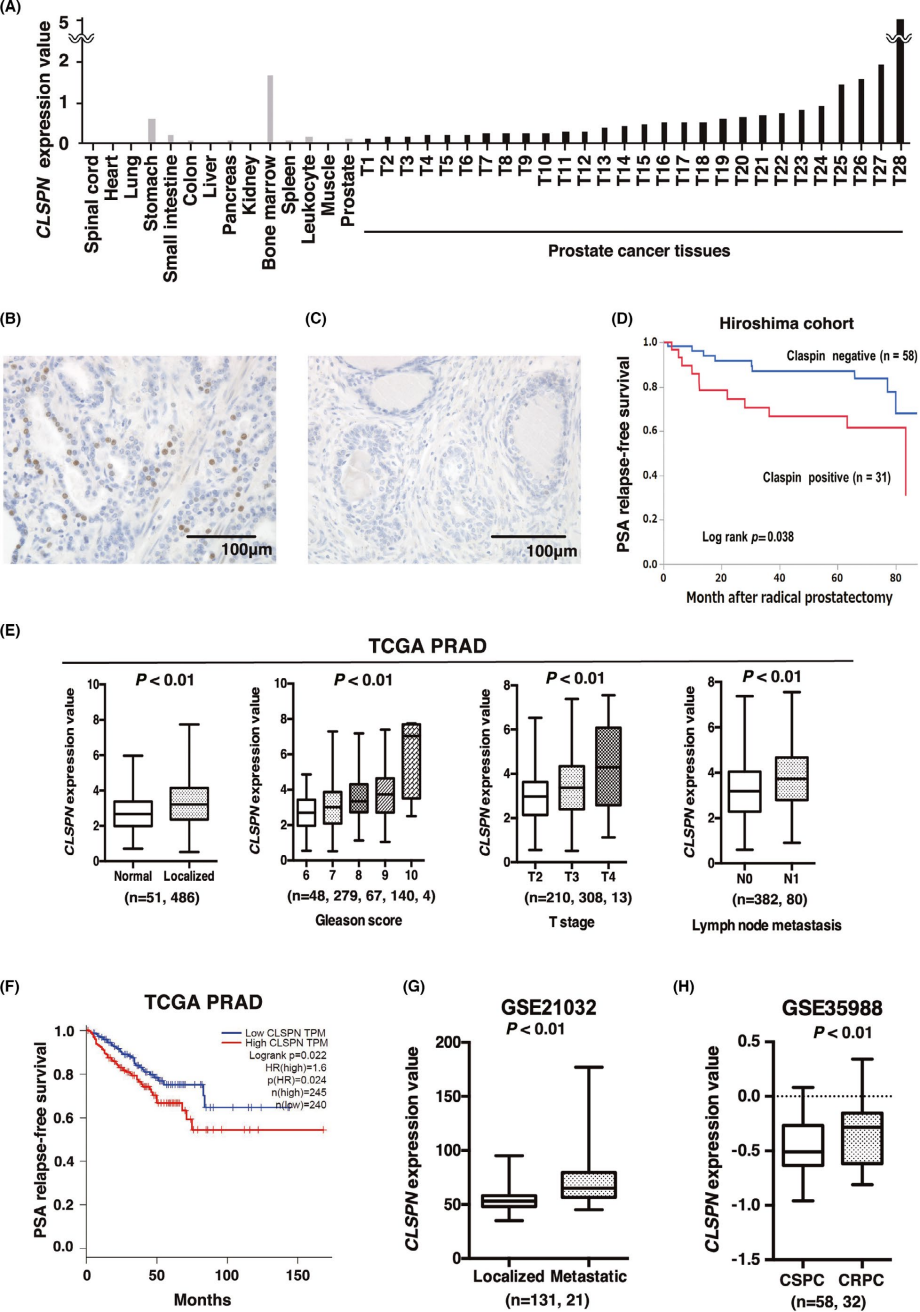
Claspin expression analysis in PCa. (A) qRT‐PCR analysis of *CLSPN* in 14 kinds of normal tissue and 28 PCa tissues. (B) Claspin staining was observed in the nuclei of PCa cells (anti‐claspin antibody IHC staining, original magnification, ×400). Scale bar = 100 μm. (C) Claspin expression in non‐neoplastic prostate (anti‐claspin antibody IHC staining, original magnification, ×400). Scale bar = 100 μm. (D) Kaplan–Meier plot of PSA relapse‐free survival of patients with PCa from our 89 in‐hospital samples. (E) Comparisons of *CLSPN* mRNA expression levels between Gleason scores, T stages, and lymph node metastasis are displayed as a box plot from TCGA datasets. (F) Kaplan–Meier plot of PSA relapse‐free survival of patients with PCa from the TCGA dataset. (G, H) Comparison of *CLSPN* mRNA expression levels between localized and metastatic tumors or castration‐sensitive prostate cancer (CSPC) and CRPC in multiple GSE datasets

We performed qRT‐PCR to confirm whether the *CLSPN* gene is cancer specific in 14 types of normal tissue and 28 PCa tissues obtained from radical prostatectomy specimens. *CLSPN* expression was detected at low levels in various normal organs including in the non‐neoplastic prostate tissue. However, it was observed to be 1.1–38.4‐fold higher in the PCa than the non‐neoplastic prostate tissue (Figure 1A).

IHC was performed in the 89 cases to examine the distribution and localization of claspin in the PCa and the non‐neoplastic prostate. In total, claspin was stained in the nucleus in 31 (35%) of the 89 PCa cases (Figure 1B). In the non‐neoplastic prostate, claspin staining was either faint or absent (Figure 1C). Next, we analyzed the relationship between claspin expression and various clinicopathological characteristics. Claspin expression was associated with higher Gleason score (*p* = 0.015), venous invasion (*p* = 0.015), and perineural invasion (*p* = 0.050) (Table 1) but was not associated with age, initial PSA, pT stage, and lymphatic invasion.

**TABLE 1:** Relationship between claspin expression and clinicopathologic characteristics in 89 prostate cancer cases

	claspin expression		*p*‐value*
	Positive (*n* = 31) (%)	Negative (*n* = 58) (%)	
Age			
<70 (*n* = 58)	20 (34%)	38 (66%)	N.S.
≥70 (*n* = 31)	11 (35%)	20 (65%)	
Initial PSA (ng/ml)			
<10 (*n* = 61)	19 (31%)	42 (69%)	N.S.
≥10 (*n* = 28)	12 (43%)	16 (57%)	
Gleason score			
6/7 (*n* = 77)	23 (30%)	54 (70%)	0.015
8/9/10 (*n* = 12)	8 (67%)	4 (33%)	
pT stage			
pT1/T2 (*n* = 77)	26 (34%)	51 (66%)	N.S.
pT3/T4 (*n* = 12)	5 (42%)	7 (58%)	
Lymphatic invasion			
Negative (*n* = 83)	28 (34%)	55 (66%)	N.S.
Positive (*n* = 6)	3 (50%)	3 (50%)	
Venous invasion			
Negative (*n* = 77)	23 (30%)	54 (70%)	0.015
Positive (*n* = 12)	8 (67%)	4 (33%)	
Perineural invasion			
Negative (*n* = 57)	24 (42%)	33 (58%)	0.050
Positive (*n* = 32)	25 (78%)	7 (22%)	

Abbreviations: N.S., not significant; PSA, prostate‐specific antigen.

* *p*‐values were calculated with Fisher's exact test.

As no patients died during our observation period, we performed a Kaplan–Meier analysis to investigate the association between claspin expression and the prognosis for biochemical recurrence in PCa. Claspin‐positive cases were associated with PSA relapse‐free survival in the 89 PCa cases (*p* = 0.038, log‐rank test; Figure 1D).

To evaluate the potential of claspin expression as a prognostic marker for PSA relapse‐free survival, we performed univariate and multivariate Cox proportional hazards analyses. In the univariate analysis, initial PSA, Gleason score, and perineural invasion were associated with PSA relapse‐free survival. In the multivariate models, initial PSA and perineural invasion were independent prognostic indicators of relapse, but claspin expression was not. However, high claspin expression tended to worsen the PSA relapse‐free prognosis (*p* = 0.052, log‐rank test; Table 2).

**TABLE 2:** Univariate and multivariate Cox regression analyses of PSA relapse‐free survival

	Univariate analysis		Multivariate analysis	
	HR (95% CI)	*p*‐value	HR (95% CI)	*p*‐value
Age				
<70	1 (Ref.)			
≥70	1.02 (0.38–2.49)	0.971		
Initial PSA (ng/ml)				
<10	1 (Ref.)		1 (Ref.)	0.009
≥10	3.65 (1.50–9.35)	0.004	3.48 (1.38–9.30)	
Gleason score				
6/7	1 (Ref.)		1 (Ref.)	0.113
8/9/10	3.28 (1.15–8.29)	0.028	3.00 (0.76–10.35)	
pT stage				
pT1/T2	1 (Ref.)		1 (Ref.)	0.341
pT3/T4	1.23 (0.28–3.68)	0.752	0.51 (0.10–1.95)	
Lymphatic invasion				
Negative	1 (Ref.)		1 (Ref.)	0.525
Positive	4.18 (0.96–12.81)	0.055	1.87 (0.23–9.53)	
Venous invasion				
Negative	1 (Ref.)		1 (Ref.)	0.877
Positive	1.75 (0.50–4.84)	0.346	1.12 (0.22–4.23)	
Perineural invasion				
Negative	1 (Ref.)		1 (Ref.)	0.050
Positive	2.81 (1.07–7.53)	0.037	2.78 (1.00–8.06)	
Claspin expression				
Negative	1 (Ref.)		1 (Ref.)	0.052
Positive	2.48 (1.02–6.18)	0.045	2.51 (0.99–6.54)	

Abbreviations: HR, hazard ratio; Ref., reference.

To confirm the above findings, we checked the expression of *CLSPN* in various cancer datasets. From the TCGA data set, the *CLSPN* expression level increased significantly with increasing Gleason Scores and T stages and was higher in the group with lymph node metastases than that without metastases (Figure 1E). The PSA relapse‐free survival curve was similar to our above data (*p* = 0.022, log‐rank test; Figure 1F). We observed that the *CLSPN* expression level was increased in metastatic PCa (GSE21032) (Figure 1G) and CRPC (GSE35988) (Figure 1H).

### 3.2 *CLSPN* expression in neuroendocrine prostate cancer

**FIGURE 2:**
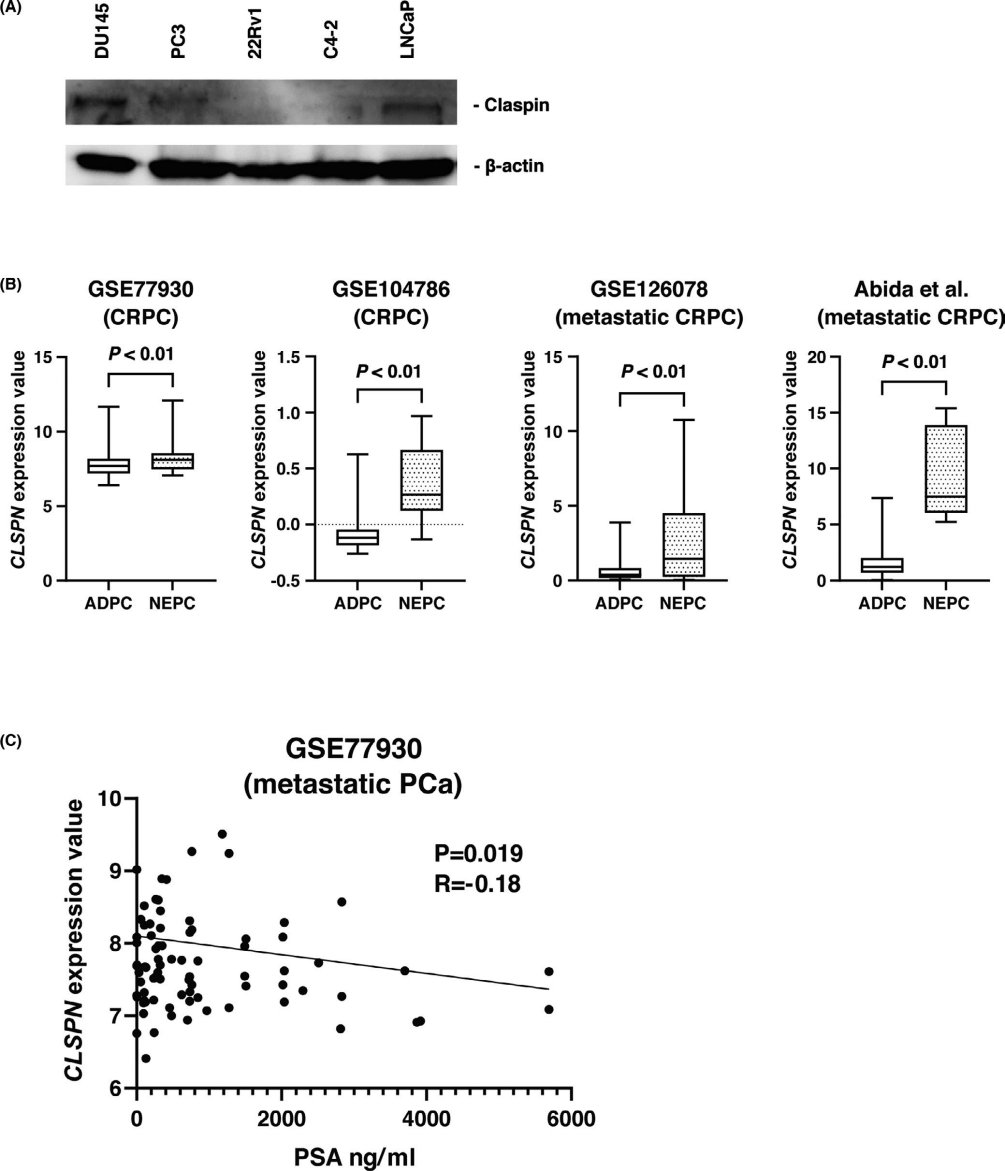
The *CLSPN* expression in neuroendocrine prostate cancer. (A) Western blot analysis of claspin in PCa cell lines. The band was detected at approximately 175 kDa. β‐Actin was used as a loading control. (B) Comparison of *CLSPN* mRNA expression levels between prostate adenocarcinoma (ADPC) and neuroendocrine prostate cancer (NEPC) in multiple GSE datasets of CRPC. (C) Association of *CLSPN* expression level and PSA level in GSE77930

Western blot analysis using five PCa cell lines revealed high claspin expression in the PC3 and DU145 cell lines, which are characterized as AR‐ and PSA‐negative cell lines^38^ (Figure 2A). Therefore, we analyzed both the association of claspin with AR and neuroendocrine differentiation and the relationship between claspin expression and AR expression in the 89 PCa cases by IHC. Claspin expression was not associated with AR expression (Table 3). Next, we checked the expression of *CLSPN* in four public datasets related to neuroendocrine prostate cancer (NEPC), including metastatic CRPC (GSE77930, GSE104786, GSE126078, and the dataset of Abida et al.). We observed that *CLSPN* expression increased in NEPC compared with that in conventional prostate adenocarcinoma (ADPC) (Figure 2B). In addition, we found a slight inverse correlation of *CLSPN* expression with the PSA level in GSE77930 (*p* = 0.019, *R* = −0.18, Figure 2C).

**TABLE 3:** Immunohistochemical relationship between claspin and androgen receptor expression in 89 prostate cancer cases

	claspin expression		*p*‐value*
	Positive (*n* = 31) (%)	Negative (*n* = 58) (%)	
AR			
Negative (*n* = 6)	3 (50%)	3 (50%)	N.S.
Positive (*n* = 83)	28 (34%)	55 (66%)	

Abbreviations: AR, androgen receptor; N.S., not significant.

* *p*‐value was calculated with Fisher's exact test.

### 3.3 Effect of claspin inhibition on cell proliferation

**FIGURE 3:**
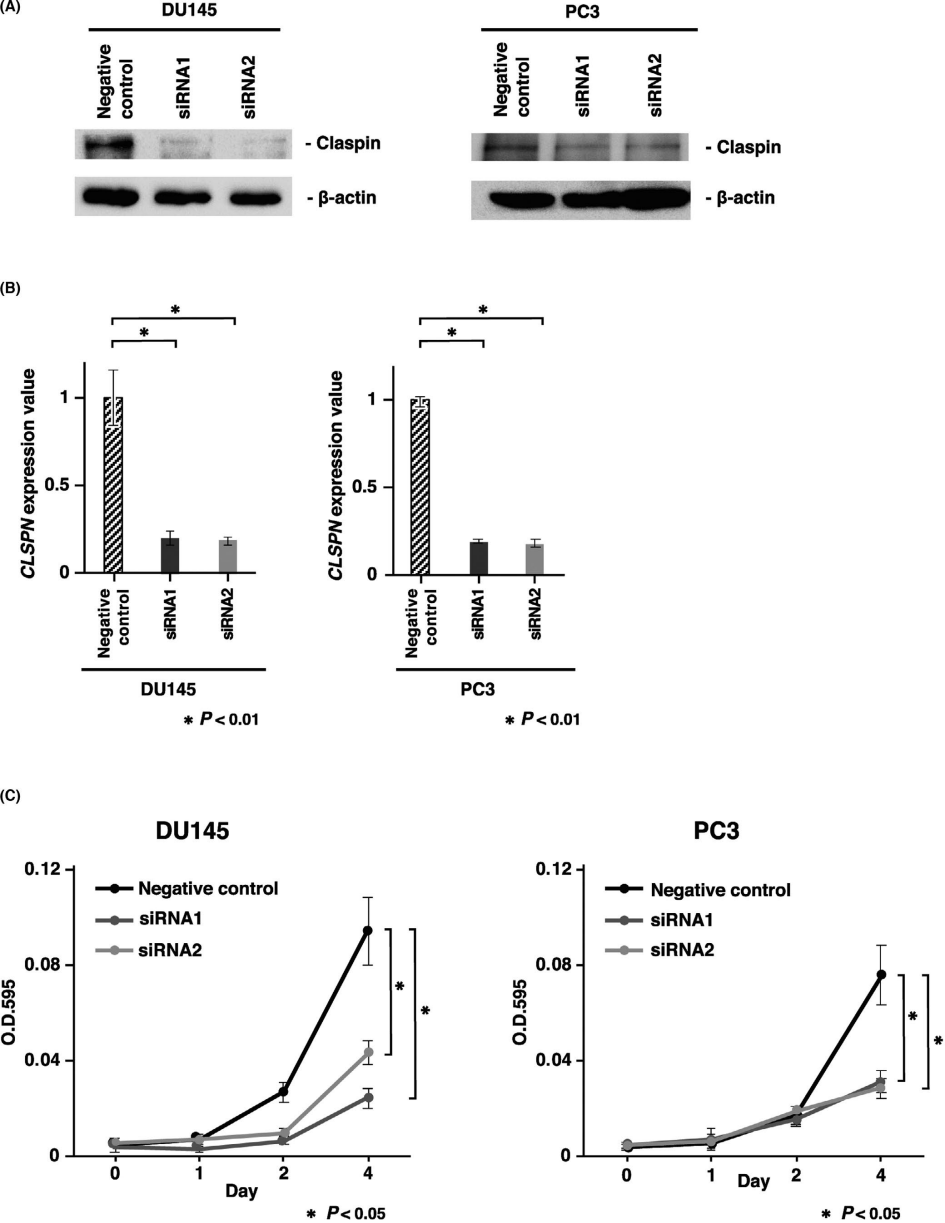
Effect of claspin inhibition on cell proliferation. (A) Western blot analysis of claspin in DU145 and PC3 cells transfected with *CLSPN* or negative control siRNAs. (B) qRT‐PCR analysis of *CLSPN* in DU145 and PC3 cells transfected with *CLSPN* or negative control siRNAs. (C) Effects of claspin knockdown on DU145 and PC3 cell proliferation. An MTT assay assessed cell proliferation at 1, 2, and 4 days after seeding on 96‐well plates. Bars and error bars indicate the mean and S.D., respectively

The biological function of claspin has never been reported in PCa cells. We next investigated the effect of claspin knockdown on cell proliferation in DU145 and PC3 cells using siRNA targeting CLSPN. Claspin expression was suppressed by treatment with siRNA1 and siRNA2, as confirmed by Western blot and qRT‐PCR (Figure 3A,B). Cell proliferative ability analyzed by MTT assay was significantly reduced in *CLSPN*‐knockdown PCa cells relative to negative control siRNA‐transfected PCa cells (Figure 3C).

### 3.4 Association of claspin inhibition with DNA damage and resistance to docetaxel

**FIGURE 4:**
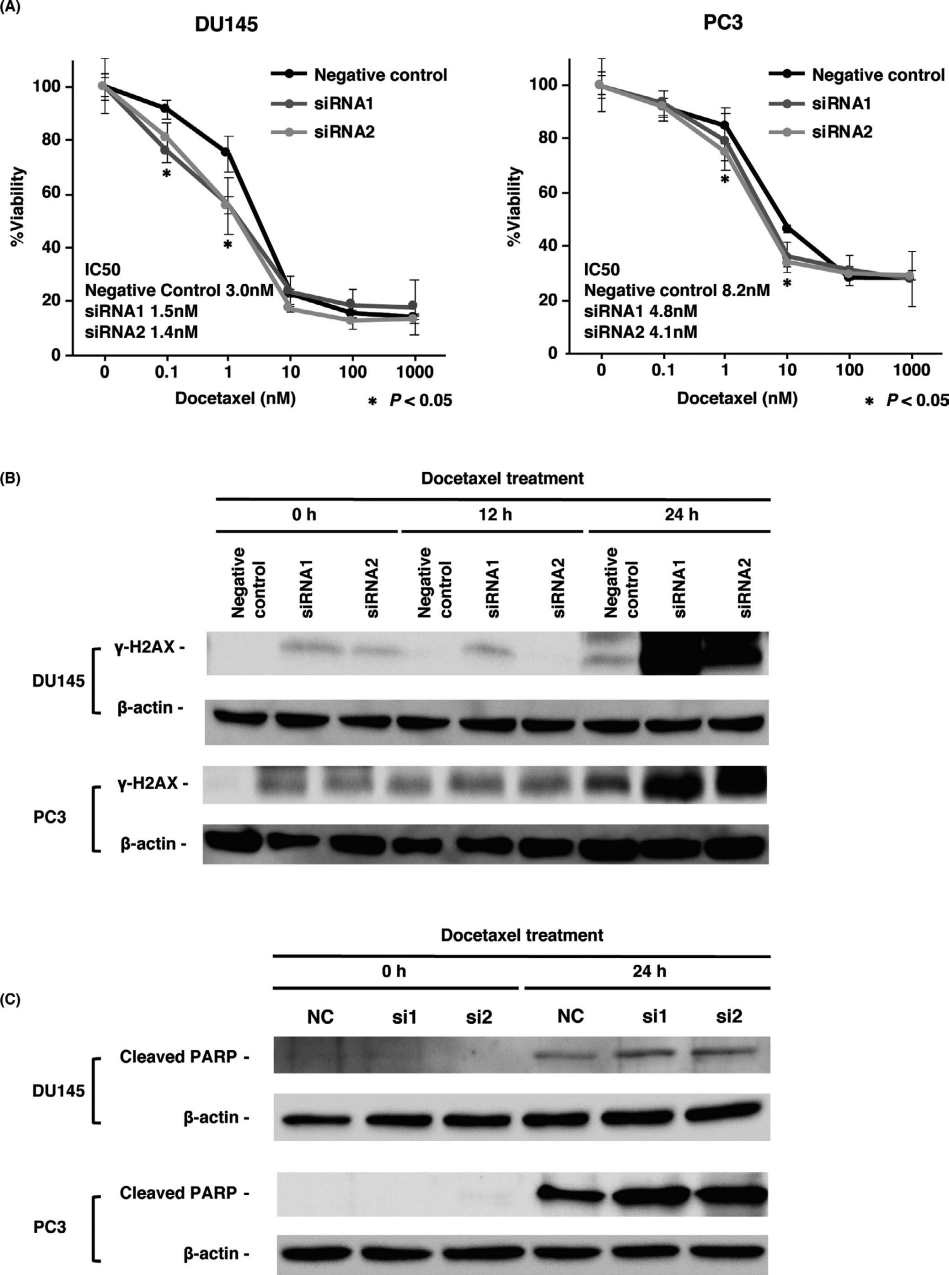
Association of claspin inhibition with DNA damage and resistance to docetaxel (DTX). (A) Dose‐dependent effect of DTX on the viability of DU145 and PC3 cells analyzed by MTT assay. Bars and error bars indicate the mean and S.D., respectively. (B) Western blot analysis of γ‐H2AX expressions in DU145 and PC3 cells transfected with *CLSPN* or negative control siRNAs after 24 h of treatment with DTX. (C) Western blot analysis of cleaved PARP expressions in DU145 and PC3 cells transfected with *CLSPN* or negative control siRNAs after 24 h of treatment with DTX

It is well known that the radio‐chemotherapy resistance of CRPC is associated with DDR pathways, such as the homologous recombinational repair genes BRCA1 and BRCA2 and the DNA damage checkpoint activator ATM.^39^, ^40^, ^41^ It was estimated that claspin was also involved in DTX resistance because of its role in DDR. MTT assays were performed to measure the cell viability of *CLSPN* siRNA‐ and negative control siRNA‐transfected DU145 cells and PC3 cells under various concentrations of DTX for 24 h. The IC50 values of the negative control siRNA‐, *CLSPN* siRNA1‐, and *CLSPN* siRNA2‐transfected DU145 cells were 3.0 nM, 1.5 nM, and 1.4 nM, respectively, whereas those of the negative control siRNA‐, *CLSPN* siRNA1‐, and *CLSPN* siRNA2‐transfected PC3 cells were 8.2 nM, 4.8 nM, and 4.1 nM, respectively. The IC50 values of the *CLSPN* siRNA1‐ and *CLSPN* siRNA2‐transfected DU145 and PC3 cells were significantly lower than that of negative control siRNA‐transfected cells (Figure 4A). Using γ‐H2AX as a marker of DNA damage and cleaved PARP as a marker of apoptosis, cytotoxicity against DTX was examined in DU145 and PC3 cells. Considering the IC50 obtained by the MTT assay mentioned above, we used the DTX concentrations of 1.5 nM for DU145 and 5.0 nM for PC3. γ‐H2AX and cleaved PARP expressions in siRNA1‐ and siRNA2‐transfected DU145 and PC3 cells were evidently higher than that of the control after 24 h of treatment with DTX (Figure 4B,C).

### 3.5 Effect of claspin inhibition on AKT, ERK, and ATR‐CHK1 pathways

**FIGURE 5:**
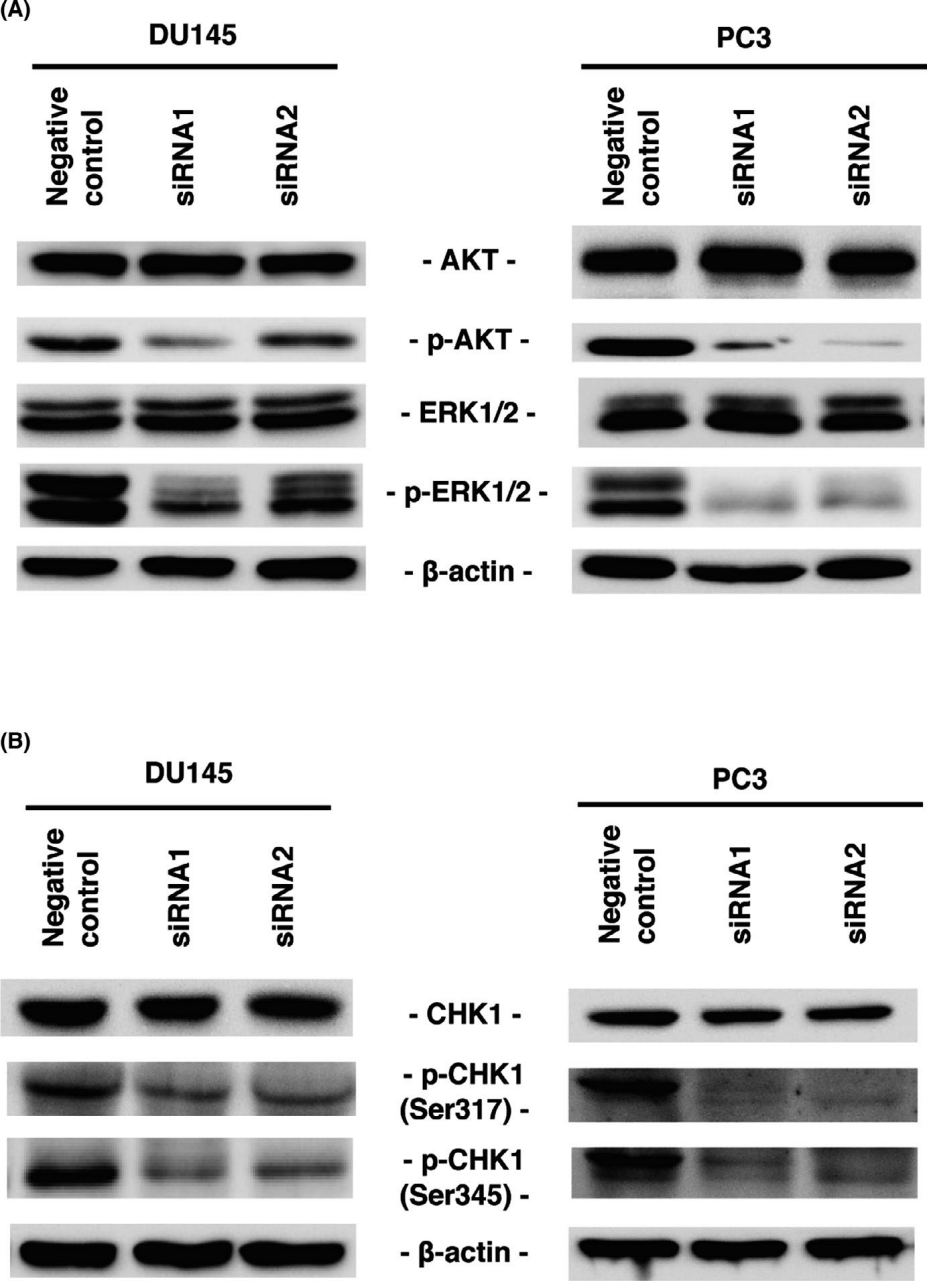
Effect of claspin inhibition on Akt, Erk, and ATR‐CHK1 pathways. (A) Western blot analysis of claspin, Akt, p‐Akt, Erk1/2, and p‐Erk1/2 in DU145 and PC3 cells transfected with *CLSPN* or negative control siRNAs. (B) Western blot analysis of claspin, CHK1, and p‐CHK1 in DU145 and PC3 cells transfected with *CLSPN* or negative control siRNAs

The importance of the PTEN‐AKT pathway is known in PCa, and it has been attracting attention as a future therapeutic target.^42^ The MEK/ERK pathway may also be involved in PCa docetaxel resistance and further research is needed.^43^, ^44^ What is more, our previous study on renal cell carcinoma showed that claspin was involved in Akt and Erk1/2 phosphorylation.^21^ Therefore, we analyzed the effect of claspin inhibition on the Akt and Erk pathways. The levels of p‐Akt and p‐Erk were lower in the *CLSPN* siRNA1‐ and siRNA2‐transfected DU145 and PC3 cells than those in the control cells (Figure 5A). These results suggest that claspin was involved in the Akt and Erk pathways. One of the roles of claspin is to activate the ATR‐CHK1 pathway,^45^ so we examined the effect of claspin inhibition on the ATR‐CHK1 pathway in PCa. The levels of phosphorylated CHK1 (p‐CHK1) (Ser317) and p‐CHK1 (Ser345) were lower in the *CLSPN* siRNA1‐ and siRNA2‐transfected DU145 and PC3 cells than those in the control cells (Figure 5B).

### 3.6 Effect of claspin inhibition on docetaxel‐resistant DU145 cell line

**FIGURE 6:**
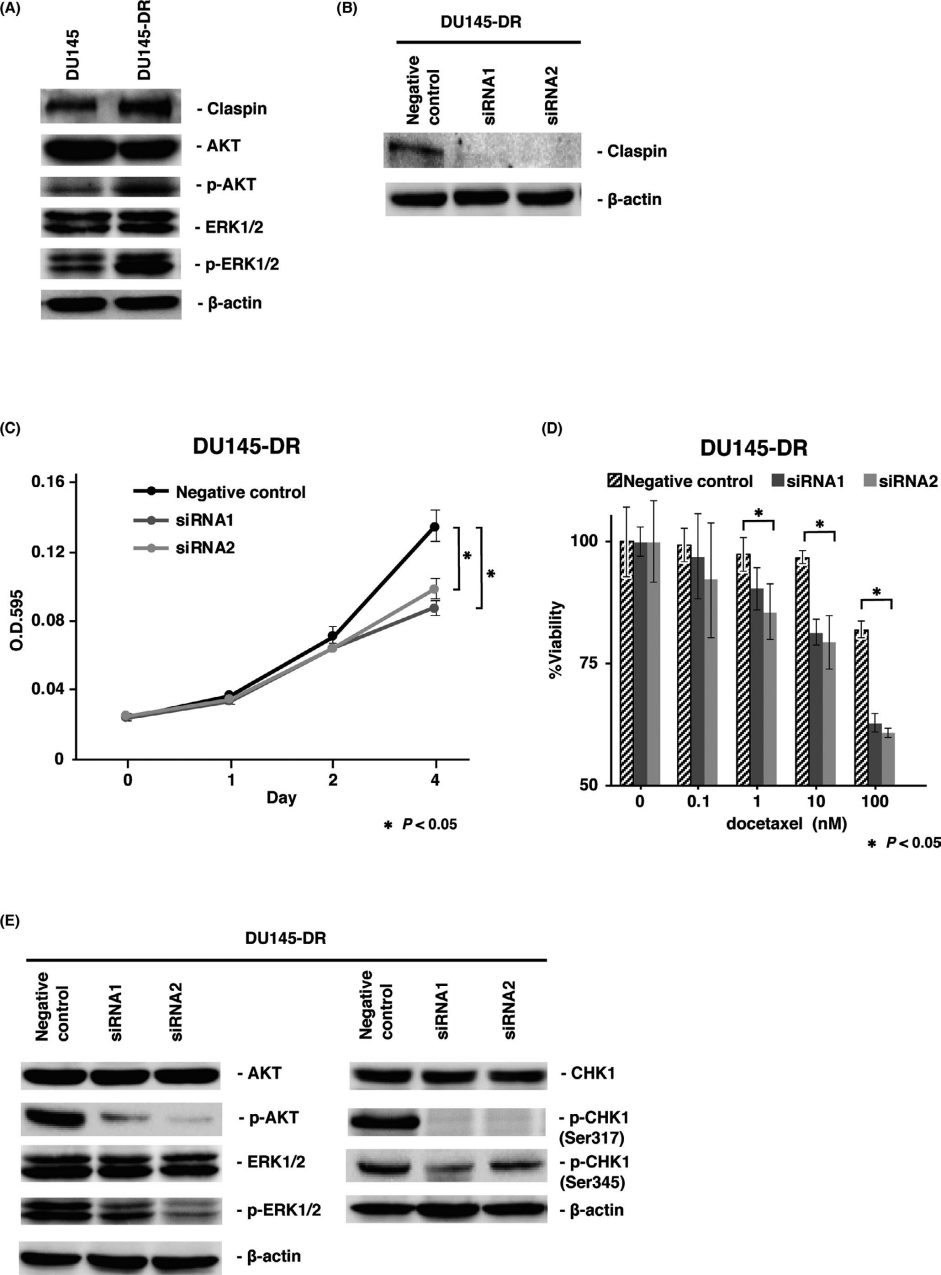
Effect of claspin inhibition on the docetaxel (DTX)‐resistant DU145 (DU145‐DR) cell line. (A) Western blot analysis of claspin, Akt, p‐Akt, Erk1/2, and p‐Erk1/2 in DU145 and DU145‐DR cells transfected with *CLSPN* or negative control siRNAs. (B) Western blot analysis of claspin, CHK1, and p‐CHK1 in DU145‐DR cells transfected with *CLSPN* or negative control siRNAs. (C) Effects of claspin knockdown on DU145‐DR cell proliferation that was assessed by an MTT assay. (D) Dose‐dependent effect of DTX on the viability of DU145‐DR cells analyzed by MTT assay. Bars and error bars indicate the mean and S.D., respectively. (E) Western blot analysis of claspin, Akt, p‐Akt, ERK1/2, p‐ERK1/2, CHK1, and p‐CHK1 in DU145‐DR cells transfected with *CLSPN* or negative control siRNAs

We analyzed the effect of claspin knockdown in the DU145‐DR (DTX‐resistant) cell line to confirm claspin involvement in DTX resistance. The expressions of claspin, p‐Akt, and p‐Erk were far more upregulated in the DU145‐DR cells than those in the parental DU145 cells (Figure 6A). Claspin expression was suppressed more in the *CLSPN* siRNA1‐ and siRNA2‐transfected DU145‐DR cells than that in the control cells (Figure 6B). The results from the MTT assay showed that cell proliferative ability was significantly reduced in the *CLSPN*‐knockdown DU145‐DR cells relative to the negative control siRNA‐transfected DU145‐DR cells (Figure 6C). Furthermore, *CLSPN*‐knockdown DU145‐DR cells significantly enhanced DTX sensitivity compared to the control cells (Figure 6D). To check the knockdown effects downstream of claspin, we studied the cell proliferation and ATR‐CHK1 pathways. The levels of phosphorylated Akt, Erk1/2, CHK1 (Ser317), and CHK1 (Ser345) were lower in the *CLSPN* siRNA1‐and siRNA2‐transfected DU145‐DR cells than those in the control cells (Figure 6E).

## 4 DISCUSSION

DDR pathways have been attracting attention as new therapeutic targets.^40^ Indeed, a recent clinical trial reported that treatment with olaparib, a PARP inhibitor, led to a high response rate in CRPC patients with DDR alterations.^46^ Claspin plays an essential role in the response to DNA damage and to replication stress.^9^ In the present study, qRT‐PCR showed that *CLSPN* expression was higher in PCa tissues than that in normal tissues. IHC analysis revealed that claspin expression was increased in PCa compared with that in non‐neoplastic prostate. Furthermore, claspin knockdown significantly reduced cell proliferation and retrieved DTX resistance in PCa cell lines. Collectively, these results indicate that claspin may be a promising therapeutic target with fewer side effects than current treatments.

The importance of DTX is increasing because upfront DTX with ADT has been shown to improve survival outcomes in metastatic castration‐sensitive prostate cancer (CSPC).^47^ In other words, in recent years, we have been able to administer DTX even to CSPC patients before their cancer changes to CRPC. Although various DTX resistance mechanisms have been mentioned, their details remain to be established. Long‐term replication stress caused by the conventional use of DTX may lead tumor cells to develop resistance to DTX.^48^ Therefore, the present study investigated the involvement of claspin in DTX resistance. We found that claspin expression was upregulated much more in DU145‐DR cells than it was in the parental DU145 cells. Furthermore, *CLSPN* knockdown enhanced DTX sensitivity in the DU145, PC3, and DU145‐DR cells. Claspin may be a molecule that partly explains the mechanism of DTX resistance in PCa. This is the first report to address the involvement of claspin in DTX resistance.

Several other reports have shown that radiation therapy or chemotherapy for cancer cells with defective DDR pathways may be more effective due to the reduction of the DNA damage response.^26^, ^49^, ^50^, ^51^ Cancer cells commonly rely more on the ATR‐CHK1 pathway than the ATM‐CHK2 pathway for survival after DNA damage. Claspin is a critical protein for the ATR‐CHK1 pathway, so cancer cells may more sensitive under the inhibition of claspin.^52^, ^53^, ^54^, ^55^, ^56^, ^57^ In the present study, we showed that claspin knockdown blocked CHK1 phosphorylation in PCa cell lines. This inhibition increased replication stress, failed to promote DDR, and may have enhanced the cytotoxic activity of DTX and led to apoptosis.

In general, PCa gradually becomes independent of AR signaling as a mechanism of treatment resistance.^58^ CRPC is still dependent on AR signaling through acquired AR gene mutation or other means to re‐activate the AR.^59^ However, NEPC is an AR‐negative and PSA‐independent small cell carcinoma that results from ADPC histological transformation.^34^ In our study, Western blotting showed that claspin expression was not detected in 22Rv‐1 and C4‐2 cell lines but in PC3 and DU145 cell lines. What is more, in silico analysis showed that *CLSPN* expression was higher in NEPC than that in ADPC and was slightly inversely correlated with PSA level in the metastatic PC datasets. These results indicate that claspin might be involved in neuroendocrine differentiation. However, the claspin expression was detected in AR‐positive LNCaP cells. Recent studies have stressed that the transition from ADPC to NEPC is a complicated process because NEPC includes heterogeneous phenotypes.^60^, ^61^, ^62^ Although it remains unclear whether claspin interacts with AR and PSA levels during the transition from ADPC to NEPC phenotype, claspin might play an essential role in the progression to NEPC.

This study has some limitations. First, we performed the retrospective analysis of claspin expression in a small sample of cases and the samples were not derived from CRPC patients but CSPC patients. Therefore, a study with a larger number of CRPC patients and prospective analysis will verify the current findings. Second, our data lack any functional assays to confirm the effects of claspin on DNA damage and apoptosis. Further analysis using apoptosis assays including flow cytometry and comet assays could support our data. Third, we used PCa cell lines to show the involvement of claspin in DTX resistance in vitro. Further studies using claspin inhibitors with CRPC against DTX resistance in vivo could support the potential of claspin inhibitors in treating PCa patients.

In conclusion, we showed with IHC that claspin is highly expressed in PCa but that its expression is low in normal cells. Claspin overexpression was associated with poor PSA relapse‐free survival and was upregulated in DU145‐DR cells. We also found that *CLSPN* knockdown enhanced DTX sensitivity and deregulated Akt, Erk1/2, and CHK1 phosphorylation in PCa cell lines. Claspin may be a pivotal protein to improve DTX‐based chemotherapy and could potentially be a promising therapeutic target in PCa.

## CONFLICT OF INTEREST

The authors have no conflict of interest.

## Data Availability

All the data supporting the findings of this study are available from the corresponding authors on reasonable request.
